# Epidermal growth factor expression as a predictor of chemotherapeutic resistance in muscle-invasive bladder cancer

**DOI:** 10.1186/s12894-018-0413-9

**Published:** 2018-11-09

**Authors:** Ahmed M. Mansour, Mona Abdelrahim, Mahmoud Laymon, Mamdouh Elsherbeeny, Mohammed Sultan, Ahmed Shokeir, Ahmed Mosbah, Hassan Abol-Enein, Amira Awadalla, Eunho Cho, Vikram Sairam, Taeeun D. Park, Muhammad Shahid, Jayoung Kim

**Affiliations:** 1 Urology and Nephrology Center, Mansoura University, Mansoura, Egypt; 2 University of Texas Health Science Center, San Antonio, USA; 3 University of California Los Angeles, Los Angeles, CA USA; 4 University of California, Berkerly, CA USA; 5 Departments of Surgery and Biomedical Sciences, Samuel Oschin Comprehensive Cancer Institute, Cedars Sinai Medical Center, 8700 Beverly Blvd, Los Angeles, CA 90048 USA

**Keywords:** EGFR, Bladder cancer, Survival, Adjuvant chemotherapy

## Abstract

**Background:**

Epidermal growth factor receptor (EGFR) overexpression is believed to be associated with bladder cancer (BC) progression and poor clinical outcomes. In vivo studies have linked EGFR subcellular trafficking and chemo-resistance to cisplatin-based chemotherapies. This has not been studied in the clinical adjuvant setting. We aimed to investigate the prognostic significance of EGFR expression in patients receiving cisplatin-based adjuvant chemotherapy following radical cystectomy for advanced BC.

**Methods:**

The database from the Urology and Nephrology Center at Mansoura University was reviewed. BC patients who were treated with radical cystectomy and adjuvant chemotherapy for adverse pathological features or node positive disease were identified. Patients who underwent palliative cystectomy, had histological diagnoses other than pure urothelial carcinoma, or received adjuvant radiotherapy were excluded from the study. Immunohistochemical staining for EGFR expression was performed on archived bladder specimens. The following in vitro functional analyses were performed to study the relationship of EGFR expression and chemoresponse.

**Results:**

The study included 58 patients, among which the mean age was 57 years old. Majority of patients had node positive disease (*n* = 53, 91%). Mean follow up was 26.61 months. EGFR was overexpressed in 25 cystectomy specimens (43%). Kaplan-Meier analysis revealed that EGFR over-expression significantly correlated with disease recurrence (*p* = 0.021). Cox proportional hazard modeling identified EGFR overexpression as an independent predictor for disease recurrence (*p* = 0.04). Furthermore, in vitro experiments demonstrated that inhibition of EGFR may sensitize cellular responses to cisplatin.

**Conclusions:**

Our findings suggest that EGFR overexpression is associated with disease recurrence following adjuvant chemotherapy for advanced BC. This may aid in patient prognostication and selection prior to chemotherapeutic treatment for BC.

## Background

Bladder cancer (BC) is the second most common genitourinary malignancy and the fourth most common cancer in the United States (U.S.). Over an estimated $4 billion/year is spent on BC treatment annually in the U.S., making BC one of the most expensive cancer treatments to date [1–3]. Currently, BC is also the most common cancer in Egyptian males, representing about 30% of all cancer types [4]. Thus, BC is a major burden on the health services and economic resources at an international level [5]. Despite the drastic decrease in the prevalence of schistosomiasis in Egypt due to nationwide anti-bilharzial campaigns, there has been an increase in incidences of bladder urothelial carcinoma. This could possibly be due to smoking and carcinogenic chemical exposure [6, 7].

The gold standard therapy for patients with muscle-invasive bladder cancer (MIBC) is radical cystectomy with regional lymphadenectomy. Despite local aggressive therapy, nearly half of patients eventually develop metastasized tumors and, ultimately, die from the disease [8]. In an attempt to improve survival, integration of systemic chemotherapy with surgical management has been suggested to control micrometastasis [9]. However, around 40% of patients receiving neoadjuvant chemotherapy are termed “non-responders”, with a complete pathological down-staging rate of only 14–38% [10, 11]. MIBC patients who do not respond to adjuvant chemotherapy generally have a poor prognosis [12]. The incidence of BC recurrence following chemotherapy remains high with a modest survival advantage of 5–15%. Thus, there is an important and urgent need to identify prognostic marker(s) that will identify patients who are at risk and to better understand the functional contribution of potential predictive markers in aggressive BC.

Prior research has shown that epidermal growth factor receptor (EGFR) overexpression has been associated with BC progression and poor clinical outcomes [13, 14]. In vivo studies have linked EGFR subcellular trafficking and chemo-resistance in many tumor types [15, 16]. However, this has not yet been studied in the clinical adjuvant setting.

In this study, we aimed to investigate the prognostic significance of EGFR expression in patients receiving adjuvant chemotherapy. Our study was conducted on an Egyptian cohort. Our findings suggest that EGFR protein expression may be indicative of aggressive BC and these expression patterns possibly involve direct action on signaling pathways in BC cells.

## Methods

### Patients and tissue samples

All of the enrolled patients had been treated with similar or identical regimens with at least four cycles of cisplatin-based chemotherapy. Patients previously treated with radical cystectomy and had completed adjuvant chemotherapy for adverse pathological features or node positive diseases were selected. Exclusion criteria were applied to patients who underwent palliative cystectomy, those with histological diagnosis other than pure transitional cell carcinoma, and patients who received adjuvant radiotherapy. Bladder tumors were staged according to the 2002 TNM classification. Disease progression was defined as newly diagnosed distant metastases with a ≥ 20% increment increase in tumor mass following radical cystectomy. Surgical tumor tissues were macro-dissected, typically within 15 min of surgical resection. Each BC specimen was confirmed as representative by analysis of adjacent tissue in fresh frozen sections from radical cystectomy specimens.

### Reagents

Cisplatin was purchased from Sigma. Antibodies against EGFR and β-actin were obtained from Cell Signaling Technology (for Western blot analysis), Abcam (for IHC analysis) and Santa Cruz Biotechnology. The ECL detection kit was from BioRad and New England Nuclear. All other biochemical reagents were purchased from Sigma or BD Biosciences.

### Immunohistochemical staining

Immunohistochemical (IHC) analysis for EGFR expression was performed on archived bladder specimens. The relationship of EGFR expression and clinical outcomes was assessed. In vitro studies were performed to determine whether EGFR expression was associated with resistance to chemotherapeutic reagents. Paraffin blocks from 58 BC cases were used for immunohistochemical analysis. Tissue sections were cut and placed on Superfrost Plus microscope slides. Using the Benchmark XT automated immunohistochemistry stainer (Ventana Medical Systems, Inc., Tucson, AZ, USA), slides were stained following typical procedure. Detection was done using the Ventana Ultraview DAB Kit (Ventana Medical Systems).

Sections were deparaffinized using EZ Prep solution. CC1 standard (pH 8.4 buffer contained Tris/Borate/EDTA) was used for antigen retrieval. DAB inhibitor (3% H_2_O_2_, Endogenous peroxidase) was blocked for 4 min at 37 °C temperature. Sections were incubated with an anti-EGFR (Cat # ab32077, Abcam Inc., San Diego, CA, dilution 1/100) primary antibody for 40 min at 37 °C, and then incubated with a secondary antibody of Universal HRP Multimer for 8 min at 37 °C. Slides were then incubated with DAB + H_2_O_2_ substrate for 8 min, followed by hematoxylin and bluing reagent counterstain at 37 °C. Reaction buffer (pH 7.6 Tris buffer) was used as the washing solution. Staining intensity and proportion of positively-stained cells were evaluated. Staining intensity was classified as follows: none (score 0), weak (score 1), moderate (score 2) and strong (score 3). Each specimen was examined and scored separately by two pathologists, and discrepancies were discussed until agreements were reached.

### Cell culture and transfection

TCCSUP or T24 human BC cells were purchased from American Type Culture Collection (ATCC, Manassas, VA) and maintained in DMEM or RPMI1640 (Invitrogen, Carlsbad, CA) with 10% FBS and 1% Penicillin/Streptomycin at 37 °C under 5% CO_2_. The day before transfection, TCCSUP or T24 cells were trypsinized and counted. Cells were plated in 6-well plate with approximately 6.25 × 10^5^ cells per well in 2 ml of complete growth medium. When cell density reached 80–90% confluence, TCCSUP or T24 BC cells were transiently transfected with 25-50 nM of small interfering RNAs (siRNAs) targeting EGFR (SignalSilence® EGF Receptor siRNA, Cell Signaling #6482) using Lipofactamine 2000. For transfection controls, empty (Ctrl) or non-target siRNAs (siCtrl) were used.

### Cell viability assay

Experiments were performed in 6-well plates after cell density reached to about 90% (2 × 10^3^/well). TCCSUP or T24 cells were transfected with various constructs or siRNAs and cisplatin simultaneously for 48 h (siRNA added 2 h before cisplatin). Cells were then incubated with cisplatin containing serum-free medium (RPMI1640 or DMEM) for the indicated time. Cell viability was determined using MTS reagents, as instructed by the company’s protocol (Promega Corporation, Madison, WI).

### Statistical analysis

Univariate analysis with the Pearson chi-square was performed to analyze associations between strong EGFR expression and pT stage, pN stage, (N0 and greater than N0) and lymphovascular invasion. A Kaplan-Meier estimator curve with the log rank test and a Cox proportional hazard model were used to test whether observed response to chemotherapy predicted disease specific survival.

## Results

### Baseline characteristics

The study included 58 patients. The mean age of the 57 patients who received adjuvant therapy was 57 ± 6.6 years, and the mean follow-up period was 26.61 months. A majority of patients had node positive disease (*n* = 53, 91%). Forty-five patients (77%) had lymphovascular invasion. Other baseline characteristics of the patients are presented in Table 1.

**Table 1:** Baseline characteristics of the patients in this study

Variables		Incidence (SD or %)
Age mean (SD)		57 (6.6)
No. gender (%)	Male	53(91.4)
	Female	5 (8.6)
No. clinical T stage (%)	T2	7 (12.1)
	T3	30 (51.7)
	T4a	18 (31.1)
	T4b	3 (5.2)
No. pathologic T stage (%)	TIS	1 (1.7)
	T1	1 (1.7)
	T2a	7 (12.1)
	T2b	7 (12.1)
	T3a	22 (37.9)
	T3b	15 (25.9)
	T4a	5 (8.6)
No. pathologic N status (%)	N0	5 (8.6)
	N1	14 (24.1)
	N2	37 (63.8)
	N3	2 (3.4)
No. lymphovascular invasion (%)	Yes	45 (77)
	No	13 (23)
No. strong EGFR expression (%)	Yes	10 (17.2)
	No	48 (82.8)

### EGFR expression is negatively correlated with survival

**Fig. 1:**
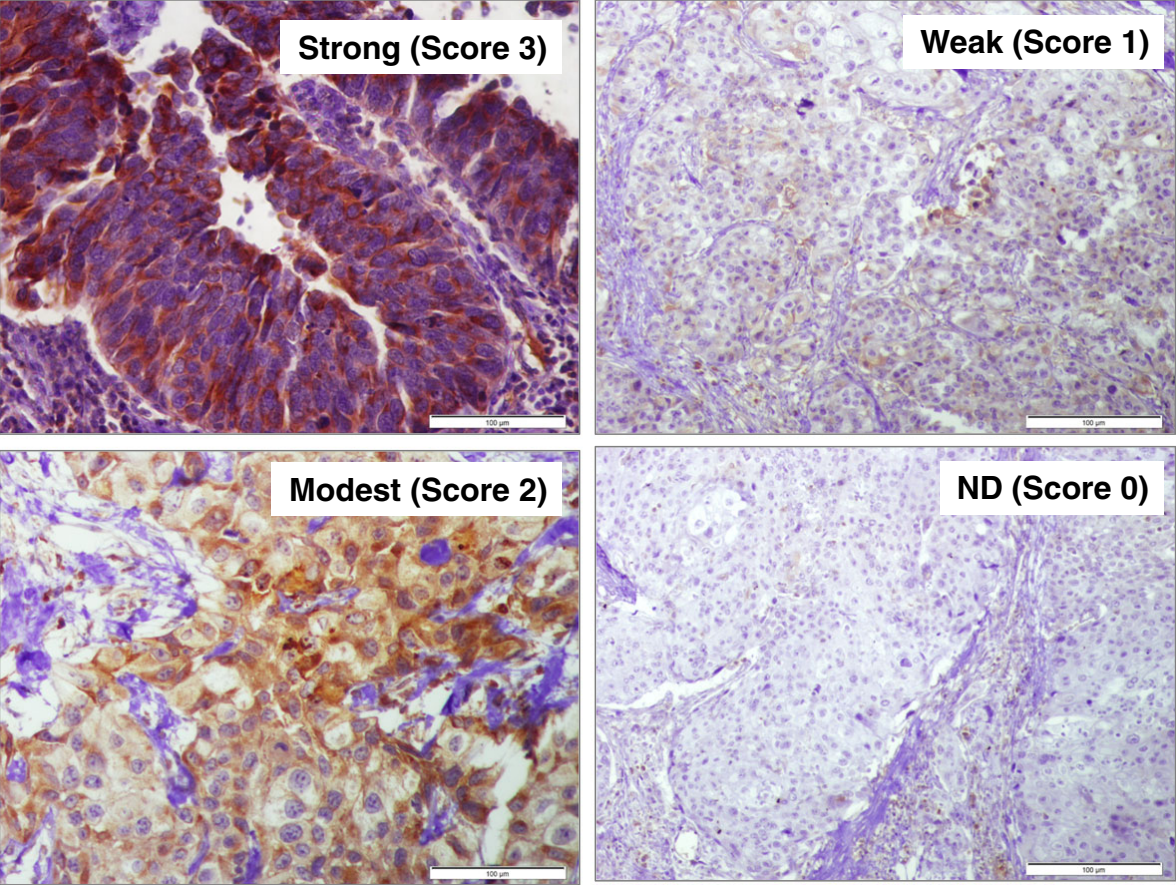
Representative figures showing IHC slides with different scores. Immunohistochemical staining for EGFR in BC tissue samples. Four representative fields are shown after IHC staining with anti-EGFR (1:1000 dilution). Negative (Score 0), weak (Score 1), intermediate (Score 2), and strong (Score 3). EGFR expression was observed in the cytoplasm, membrane, and/or nucleus in our BC specimens. The intensity of EGFR staining was often heterogeneous within the same cancer tissue.

**Fig. 2:**
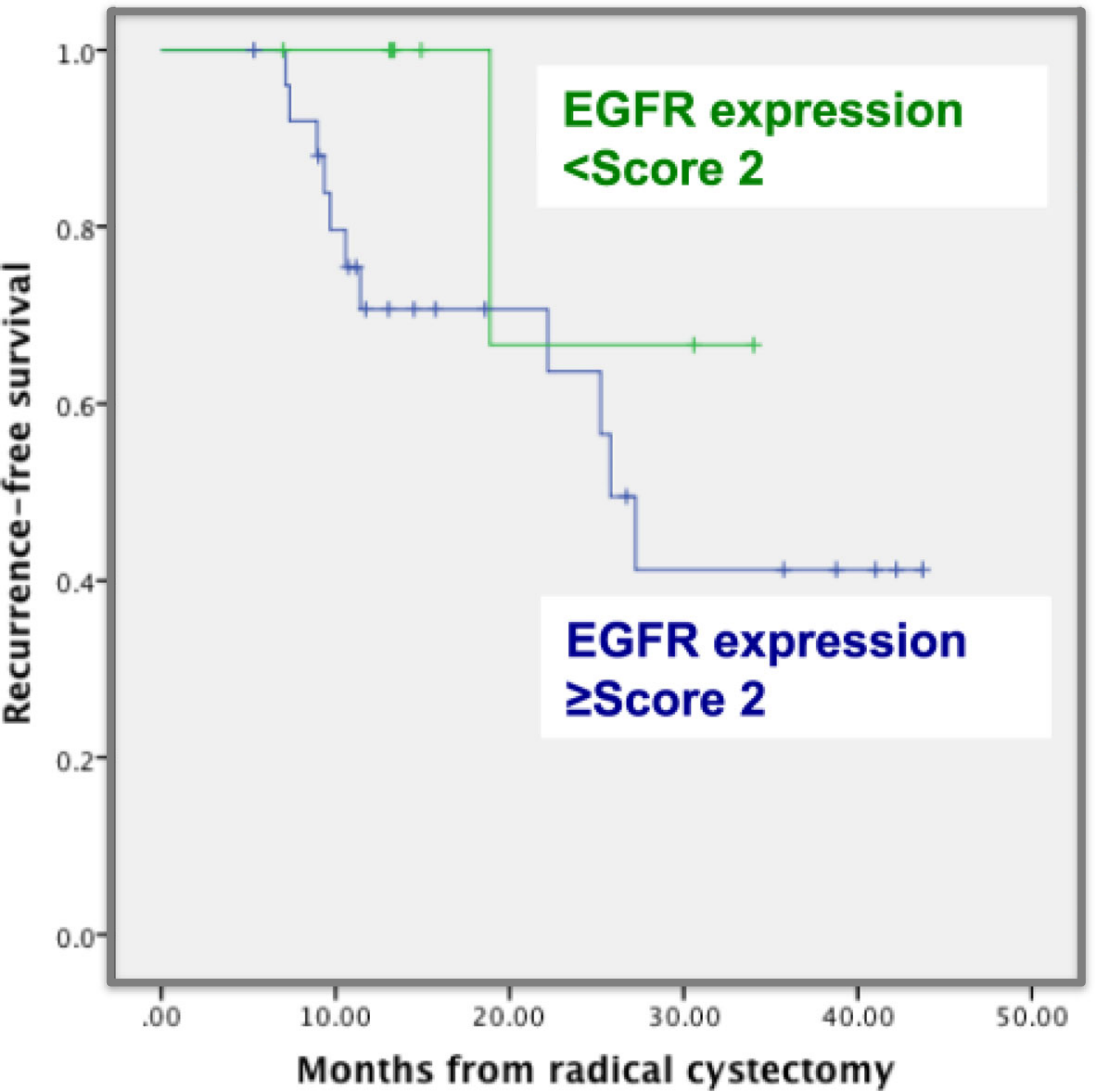
Cancer-specific survival in BC patients stratified by EGFR staining

To measure EGFR expression in our cohort, IHC analysis was performed. IHC images were scored from 0 (negative staining) to 3 (highest staining intensity). Representative images are shown in Fig. 1. Cox proportional hazard modeling identified EGFR overexpression as an independent predictor for disease recurrence (OR, 1.38 (1.201–2.744), *p* = 0.004) in the Egyptian cohort (Table 2). Kaplan-Meier analysis revealed that EGFR overexpression (score ≥ 2) significantly correlated with disease recurrence (*p* = 0.021) (Fig. 2).

**Table 2:** Cox proportional hazard model of overall survival predictors

Covariate	Univariate		Multivariate	
	HR (95% CI)	*P* value	HR (95% CI)	*P* value
Age	1.41 (1.02–2.45)	0.029	1.34 (0.94–1.77)	0.056
Sex (M vs F)	1.88 (1.06–3.34)	0.048	1.48 (0.26–1.80)	0.479
EGFR expression (strong vs negative/weak/moderate)	1.55 (1.30.-2.33)	0.002	1.38 (1.201–2.744)	0.004
Chemotherapy Regimen (MVAC vs GemCis)	1.72 (0.84–3.75)	0.159		
pT stage (T2 or less vs greater than T2)	2.88 (1.92–3.99)	0.003	3.28 (1.54–4.62)	< 0.001
pN stage (N0 vs greater than N0)	2.31 (1.88–2.93)	< 0.001	1.81 (1.23–2.74)	< 0.001

### EGFR silencing alters cell proliferation, viability and response to cisplatin-induced apoptosis

**Fig. 3:**
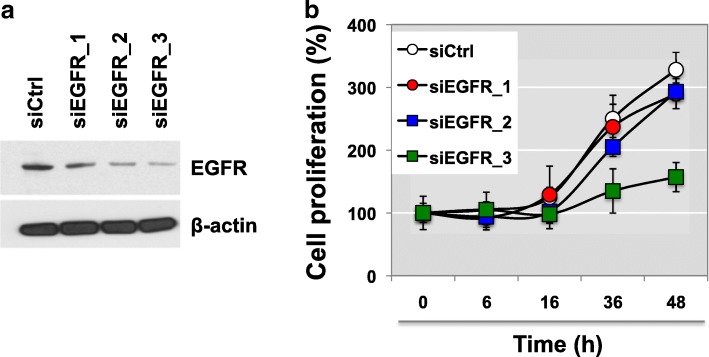
EGFR expression is associated with drug sensitivity to cisplatin treatment. **a**, **b** TCCSUP cells were transiently transfected with varying doses of siRNA against EGFR. Knockdown of EGFR with control and EGFR-targeted siRNAs shows that proliferation in TCCSUP BC cells decreases in a dose-dependent manner (siEGFR_1, siEGFR_2, siEGFR_3). Cell proliferation assay was performed at the indicated time points (0, 6, 16, 36, or 48 h after transient transfection with siRNAs) using MTT assay at the varying time points. *p < 0.05 (Student’s t-test)

We further performed loss-of-function studies on TCCSUP human BC cells to assess the biological role of EGFR. EGFR was knocked-down using iRNAs and this was subsequently confirmed via western blot analysis (Fig. 3a). Silencing of EGFR did not induce a morphological switch. However, in vitro functional analysis demonstrated that EGFR expression levels can alter cell proliferation rates in TCCSUP BC cells. A dose dependent transfection of EGFR siRNAs (siEGFR_1, _2, or _3) revealed that EGFR deficiency evoked an approximately 50% decrease in cell proliferation (Fig. 3b). This data implicates that EGFR loss as an important mechanism through which BC cells keep proliferating.

**Fig. 4:**
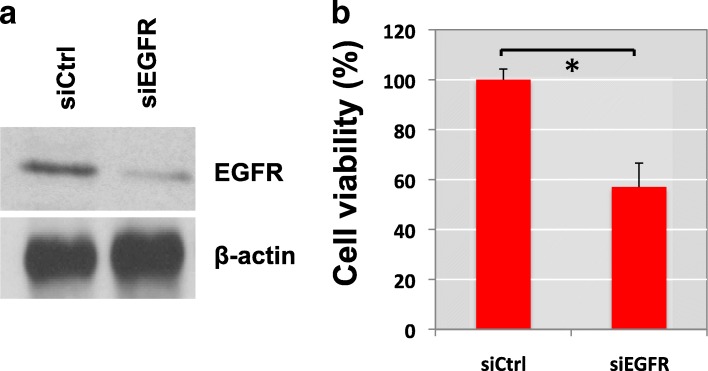
Knockdown of EGFR suppresses recovery from cisplatin treatment. **a** EGFR silenced TCCSUP cells (siEFGR) or control TCCSUP cells (siCtrl) were challenged with cisplatin treatment. TCCSUP cells were incubated with cisplatin (10 µM) and siRNAs for 48 h and then re-treated with cisplatin alone for an additional 6 h. After, the cisplatin was removed from the culture media and cells were incubated in normal growth medium for 24 h. **b** Cell viability was measured using MTT assay. Cell viability levels of three wells of transfected cells were determined. The graph was plotted as %, compared to control, no cisplatin treatment in siCtrl group (± SD). *P < 0.05 (Student’s t-test). All experiments were done in at least triplicates

We next assessed whether loss of EGFR expression can result in cell viability responses in relation to the effects of cisplatin and whether inhibition of EGFR can enhance the sensitivity of BC cells to cisplatin. We found that EGFR expression is associated with resistance to cisplatin-induced cytotoxicity. EGFR knockdown delayed cell recovery from 10 μM cisplatin treatment (Fig. 4).

Viability of control cells (siCtrl) in serum-free medium was compared with or without a challenge by 10 μM cisplatin. Cell viability assay revealed that silencing of EGFR sensitized TCCSUP BC cells to cisplatin treatment. Knockdown of EGFR was validated by western blot analysis (Fig. 4a). Cells transfected with EGFR siRNAs showed around 50% viability after cisplatin treatment compared to control TCCSUP cells (siEGFR). Removal of cisplatin from the culture medium of control cells resulted in 100% recovery of cell viabilitya (siCtrl) (Fig. 4b).

**Fig. 5:**
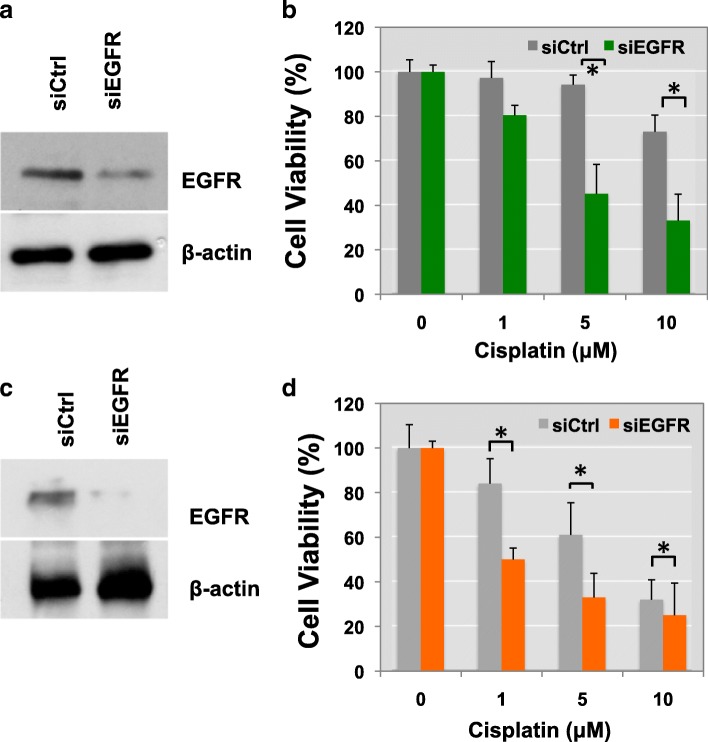
Gene silencing of EGFR enhances drug sensitivity to cisplatin treatment. (**a** and **b**, TCCSUP; **c** and **d**; T24) Transiently transfected TCCSUP (**a**) or T24 (**c**) cells with siRNA of EGFR were treated with cisplatin (0, 1, 5 and 10 μM). Cisplatin was added together with siNRA for 48 h and then re-treated with cisplatin. Cell viability was measured by MTS assay after 2 days. Overexpression of siEGFR, but not a control siRNA, in TCCSUP (**c**) or T24 (**d**) cells reduced cell proliferation (Student’s t-test, *p < 0.05)

We next sought to determine whether gene silencing of EGFR might also increase drug sensitivity to cisplatin. TCCSUP BC cells were transfected with EGFR siRNAs or control siRNAs for 48 h. Immunoblotting confirmed that EGFR expression was significantly reduced in siEGFR-transfected cells (Fig. 5a). Interestingly, loss of EGFR made TCCSUP cells more sensitive to cisplatin-induced cell apoptosis, leading to reduced cell viability. TCCSUP cells were ~2x more sensitive to 5 or 10 μM cisplatin treatments (Fig. 5b). These results were further validated in T24 BC cells (Fig. 5c-d). These findings suggest that EGFR knockdown not only suppresses the recovery of BC cells from cisplatin-reduced cell viability but also enhances the sensitivity of BC cells to cisplatin’s cytotoxicity.

## Discussion

Systemic chemotherapy is currently being used as the first line of treatment in advanced stages of BC. However, it is still unclear which group of patients will benefit and which patients will be more sensitive to cisplatin-based therapy. Our findings suggest that EGFR overexpression is associated with disease recurrence following adjuvant chemotherapy for advanced BC [17, 18]. Determining EGFR expression status may help predict prognoses and assist in deciding which patients would best benefit from adjuvant chemotherapy. Our findings also suggest that patients with higher EGFR expression may have a worse prognosis than those with little to no EGFR expression. An evaluation of intratumoral molecular marker(s) could be used to identify BC patients more likely to respond to cisplatin-based chemotherapy.

These findings align with previous studies showing that approximately 50% of BC tumor tissues overexpress EGFR and that EGFR positivity indicates more invasive cells and poor differentiation [13, 14]. However, the mechanisms through which BC tumors acquire cisplatin resistance are still elusive. Our results suggest that EGFR silencing may enhance cisplatin’s capability to shrink tumors. This observation highlights the potential of EGFR targeting strategies (e.g., kinase inhibitors or EGFR neutralizing antibodies such as gefitini, erlotinib, trastuzumab, cetuximab, matuzumutab, panitumumab et al.*)* to improve the effects of cisplatin-based chemotherapy. Recent reports have demonstrated that a subgroup of muscle-invasive bladder carcinomas with a basal-like phenotype are sensitive to EGFR kinase blockers, such as erlotinib [19, 20]. Rebouissou et al. identified a subgroup of aggressive MIBC, which shows a basal-like phenotype using their 40-gene expression classifier. In this BC subgroup, the EGFR pathway was highly activated, suggesting that anti-EGFR therapy could be used as a powerful therapeutic strategy [21, 22]. EGFR-targeted agents have only shown modest success due to acquired resistance in current ongoing clinical trials. Therefore, comprehensive clinical studies using EGFR-targeting in combination with other therapies would be more attractive.

## Conclusions

Many questions regarding EGFR silencing strategies remain unanswered. For example, what signaling cascades are modulated by high EGFR expression? How can these be regulated pharmacologically? Will BC cells obtain resistance to cisplatin? Can cells become resistant to EGFR silencing? In this study, our experimental results present EGFR as a marker of recurrence in Egyptian BC patients. Further studies are needed to better understand the regulatory mechanisms of EGFR overexpression and its downstream signaling pathways in BC, particularly in the context of squamous cell carcinoma (SCC) and transitional cell carcinoma (TCC). Our findings also suggest that elucidating some of these facets of EGFR and BC drug resistance might improve pharmacologic intervention.

## Abbreviations

BC: Bladder cancer
EGFR: Epidermal growth factor receptor
MIBC: Muscle-invasive bladder cancer
siCtrl: Control cells
siRNAs: Small interfering RNAs
